# 2475. Admissions Screening for Influenza and RSV Among a Highly Immunosuppressed Patient Population

**DOI:** 10.1093/ofid/ofad500.2093

**Published:** 2023-11-27

**Authors:** Robin T Odom, LaToya A Forrester, Angela V Michelin, Joseph M Scaletta, David K Henderson

**Affiliations:** NIH Clinical Center, Jefferson, Maryland; National Institutes of Health, Bethesda, Maryland; National Institutes of Health, Bethesda, Maryland; National Institutes of Health, Bethesda, Maryland; NIH, Bethesda, MD

## Abstract

**Background:**

During the Fall/Winter 2022-23, the U.S. experienced an early surge of Influenza and RSV cases, while SARS-CoV-2 cases persisted. With concerns of a potential “tripledemic” on the horizon, the NIH Clinical Center, a clinical research hospital with a heavily immunosuppressed patient population, implemented Influenza and RSV testing, in addition to SARS-CoV-2, for all patients on admission to facilitate early detection and prompt isolation of infected patients.

**Methods:**

Nasopharyngeal (NP) swabs (n=1213) were collected on admission from 11/2022 to 4/2023 and tested for Influenza and RSV by PCR. 1213 NP swabs from 985 unique patients were tested (some patients had multiple admissions); 1100 were tested on the Panther Fusion® SARS-CoV-2 Assay (Hologic, Inc.) and 113 using the SARS-CoV-2 RT-PCR Xpert® Xpress test. Medical chart reviews and discussions with patient care providers elucidated whether cases identified on admission were asymptomatic.

**Results:**

Of 1213 NP swabs collected from patients admitted to our hospital, 5 (0.41%) were identified with Influenza A infection, 4 (0.33%) RSV infection and no patients were identified with Influenza B. Two (0.16%) had a known history of RSV infection, 3 (0.25%) screened negative for symptoms but subsequently disclosed symptoms, 1 (0.08%) was asymptomatic on admission but disclosed a recent history of an upper respiratory infection, and 3 were asymptomatic. All infections were detected between late November 2022 to mid-January 2023, when community transmission was high, yielding a 2-month positivity rate of 2.3% during this time of peak community spread.

**Conclusion:**

Admission surveillance for Influenza and RSV during a respiratory virus surge in our community allowed us to isolate nine patients on admission who would likely not have been appropriately isolated, as symptom screening alone is not reliable. Surveillance is important to be able to identify infected patients promptly when respiratory infections are rampant in the community, and especially for institutions serving immunosuppressed patients.

**Disclosures:**

**All Authors**: No reported disclosures

